# Reactive oxygen species limit intestinal mucosa-bacteria homeostasis in vitro

**DOI:** 10.1038/s41598-021-02080-x

**Published:** 2021-12-09

**Authors:** Joshua Luchan, Christian Choi, Rebecca L. Carrier

**Affiliations:** 1 Department of Bioengineering, Northeastern University, Boston, MA 02115 USA; 2 Department of Chemical Engineering, Northeastern University, Boston, MA 02115 USA; 3 Department of Biology, Northeastern University, Boston, MA 02115 USA

**Keywords:** Microbiome, Gastrointestinal models

## Abstract

Interactions between epithelial and immune cells with the gut microbiota have wide-ranging effects on many aspects of human health. Therefore, there is value in developing in vitro models capable of performing highly controlled studies of such interactions. However, several critical factors that enable long term homeostasis between bacterial and mammalian cultures have yet to be established. In this study, we explored a model consisting of epithelial and immune cells, as well as four different bacterial species (*Bacteroides fragilis* KLE1958, *Escherichia coli* MG1655, *Lactobacillus rhamnosus* KLE2101, or *Ruminococcus gnavus* KLE1940), over a 50 hour culture period. Interestingly, both obligate and facultative anaerobes grew to similar extents in aerobic culture environments during the co-culture period, likely due to measured microaerobic oxygen levels near the apical surface of the epithelia. It was demonstrated that bacteria elicited reactive oxygen species (ROS) production, and that the resulting oxidative damage heavily contributed to observed epithelial barrier damage in these static cultures. Introduction of a ROS scavenger significantly mitigated oxidative damage, improving cell monolayer integrity and reducing lipid peroxidation, although not to control (bacteria-free culture) levels. These results indicate that monitoring and mitigating ROS accumulation and oxidative damage can enable longer term bacteria-intestinal epithelial cultures, while also highlighting the significance of additional factors that impact homeostasis in mammalian cell-bacteria systems.

## Introduction

In recent years, the gut microbiome has become increasingly linked to a wide variety of human diseases and disorders. For example, altered microbiome phylogenetic composition has been associated with autism spectrum disorders^1^, obesity^2^, and Parkinson’s disease^3^. Crosstalk between the microbiome and epithelial and immune cells has been linked to cardiovascular disease^4^, colorectal cancer^5^, and inflammatory bowel disease^6^. The intestinal epithelium is the first cellular barrier that separates the microbiome from underlying tissue harboring immune cells, which play essential roles in the regulation of gut-microbiome interactions. Antigen presenting dendritic cells send processes between the epithelial cells to sample luminal content, including microbial factors from the microbiome, and play important roles in innate immune response and recruitment of T cells via cytokine and chemokine secretion^7–10^. Most research in this field is carried out in animal models that capture the complexity of tissue and microbiome composition, but present challenges with respect to translation to humans and invasive interrogation over time^11^. The microbiome’s impact on health and the central role that the epithelium and immune system play in protecting and responding to the microbiome motivates the development of in vitro models of the human gut microbiome-epithelium-immune axis that can supplement in vivo models to answer some biological questions.

To date, the majority of reported long-term (up to 2 weeks) co-cultures with live bacteria in contact with epithelial cells have been in microfluidic devices^12,13^, although primarily short-term (< 4 h) co-cultures have been reported in static systems^14–18^, with some recent medium-term (24 h) exceptions^19^. Notably, reports of static cultures incorporating immune components, specifically dendritic cells^15,19^ or macrophage simulating differentiated THP-1 cells^20^, and bacteria result in high accumulation of dead epithelial cells and significant increases in paracellular permeability when compared to control cultures, even when cultured with bacteria for as little as 2 h. This suggests that flow is an important factor in preserving epithelial health and barrier function. It has been proposed that flow is important for preventing the accumulation of microbial waste and microbial overgrowth, thus aiding in establishing a steady-state microenvironment^12^.

It is known that intestinal epithelial and immune cells produce anti-microbial products including reactive oxygen species (ROS) upon exposure to microbes and microbial factors^21,22^. We thus hypothesized that oxidative damage may be a limiting factor in static co-culture of microbes and intestinal epithelial and immune cells. Flow may thus promote co-culture survival through the removal and dilution of anti-microbial products including ROS, as well as non-adherent bacteria and microbial products (e.g., lipopolysaccharides) that trigger their release^12^. Here, we demonstrate that oxidative damage is elevated in a static intestinal model incorporating intestinal epithelial cells, immune cells^19,23–25^, and microbes. As the addition of an antioxidant can ameliorate damage to the epithelial monolayer induced by bacterial co-culture, oxidative damage likely plays a significant role in epithelial monolayer damage in the presence of microbes. These findings highlight the significance of ROS and oxidation in the establishment of homeostasis in intestinal models incorporating microbes and demonstrate mechanisms underlying the importance of flow in microbe-mammalian co-cultures, and are thus of broad significance in development of in vitro systems for studying microbiome-host interactions and their role in health and disease.

## Materials and methods

### Mammalian/microbial co-cultures

The epithelial-immune cultures have been previously described and characterized^19,23–25^. The epithelial cultures were composed of CBBE-1 (ATCC) enterocytes (Passage 33–50) and HT29-MTX (Sigma Aldrich) mucus producing cells (Passage 27–48) seeded on 24-well Transwells with a 0.4 um pore size PET membrane (Falcon) in a 9:1 ratio at a density of 1 × 10^5^ cells per cm^2^. Cells were cultured with 300uL and 700uL in the apical and basolateral compartments, respectively, of 1X Advanced DMEM (Gibco) supplemented with 10% heat inactivated fetal bovine serum (Atlanta Biologicals), 1X GlutaMAX (Gibco), and 1% Penicillin/Streptomycin (Gibco). Dendritic cells were differentiated from human peripheral blood mononuclear cells (STEMCELL Technologies, isolated from whole blood according to supplier protocols) for 7 days in Advanced RPMI media (Gibco) containing 35 ng/mL Interleukin-4 (Sino Biological), 10 nM retinoic acid, and 50 ng/mL GM-CSF (Gibco). After 14 days of CBBE-1/HT29-MTX epithelial culture, dendritic cells were seeded on the underside of the culture membrane at a concentration of 1 × 10^5^ cells per cm^2^ to provide the model’s immune component, and given 2 h to attach. The cell cultures were then incubated with 300 µL and 700 µL in the apical and basolateral sides, respectively, of a serum-free media formulation of 1X Advanced DMEM (Gibco) supplemented with 1X GlutaMAX, 1% Penicillin/Streptomycin (Gibco), and 0.1% Insulin, Transferrin, Selenium cocktail (Gibco) for six additional days. Twenty-four hours prior to introducing the bacteria to the mammalian cultures, the basolateral medium was replaced with 700 µL of the same serum-free DMEM-based formulation but without antibiotics, and the apical serum-free DMEM was replaced with 300 µL of Dulbecco’s phosphate buffered saline + Ca^2+^ + Mg^2+^ (PBS, Corning) buffered with 10 mM of HEPES (Gibco). Single bacterial species were grown overnight from frozen stocks in MOPS microbial media and counted via hemocytometer. The bacterial stocks were then centrifuged at 6000 rpm for 5 min, and the MOPS media replaced with the PBS-HEPES apical media formulation to achieve 6.7 × 10^4^ bacteria per mL of apical media, a physiologically accurate concentration^26^, and a multiplicity of infection of roughly 0.06 $\left( {\frac{{2 \times 10^{4} \;bacterial\;cells}}{{3 \times 10^{5} \;epithelial\;cells}}} \right)$.

The bacteria were added to the apical compartment of the Transwells and given 2 h to attach. After 2 h, all of the apical and basolateral media was removed and replaced with PBS-HEPES apical media and serum-free, antibiotic-free DMEM, respectively. Cultures were maintained at 37 C and 5% CO_2_ for 48 h. Medium in both compartments was changed every 3 h during the day, with two 9-h overnight periods, during the 48-h culture period. After 48 h, the monolayer health was examined via transepithelial electrical resistance (TEER) measurement, Lucifer Yellow permeability assay, and live/dead imaging, as described below.

Experiments to determine if N-acetyl cysteine (NAC) can improve survival of the mammalian monolayers were performed in an identical manner save for the addition of 3 mM N-acetyl cysteine in the apical media starting 24 h prior to the introduction of microbes and carrying through to the end of the experiment. Experiments conducted with and without NAC supplementation were repeated 5 times with 2 biological replicates per experiment.

### Spent media studies

To identify damaging activity of agents other than live, active bacteria in the co-cultures, spent media studies were conducted by exposing mammalian monolayers composed of CBBE-1, HT29-MTX, and human dendritic cells prepared as described above to the collected apical media from the 2, 17, 26, and 50-h time points of the co-culture experiments. The spent media samples were sterilized by centrifugation at 6000 rpm for 5 min to pellet any microbes or cellular debris, followed by filtration through 0.22 µm mesh filters, and stored at -80 C. The sterile spent media was then added to the apical compartment of the CBBE-1/HT29-MTX cultures prepared as described above, with the basolateral compartments containing fresh serum-free DMEM. The cultures were exposed to the spent media for 24 h, then examined via TEER measurement, Lucifer Yellow permeability assay, and live/dead staining, as described below.

Experiments to determine if N-acetyl cysteine (NAC) can improve survival of the mammalian monolayers upon exposure to spent media were performed in an identical manner using *E. coli* MG1655 spent media supplemented with the addition of 3 mM of NAC. All spent media experiments were conducted with 3 biological replicates for each group.

### TEER measurements

TEER measurements were performed using a World Precision Instruments EVOM2 epithelial volt/ohm meter and a World Precision Instruments Endohm cell culture cup. The EVOM2 was calibrated using the reference electrode before every set of measurements, and the Endohm was sterilized between measurements per the manufacturer instructions. Measurements were taken within 5 min of removing the cultures from the incubator to minimize the influence of temperature change^27^.

### Lucifer yellow permeability

Prior to the addition of the microbes or spent media, and again at the conclusion of the experiment, a 100 µM Lucifer Yellow solution in apical media was added to the apical compartment and incubated for 1–2 h. After incubation, samples of the basolateral media were collected and their fluorescence intensities measured using a fluorescence plate reader with excitation at 428 nm and detection at 536 nm. The fluorescence intensity was then used to calculate the concentration of Lucifer Yellow using a standard curve, and the apparent permeability was calculated according to the equation $P_{app} = \frac{V}{A*Ci}*\frac{Cf}{T}$ where V is the volume of the basolateral compartment in mL, A is the area of the cell layer in cm^2^, Ci is the initial concentration of the Lucifer Yellow added in µM, Cf is the final concentration of Lucifer Yellow in the basolateral compartment after incubation, and T is the time of incubation in seconds.

### Live cell staining

Live cell staining was carried out according to the protocol for the Invitrogen LIVE/DEAD Viability/Cytotoxicity Kit for mammalian cells. In brief, an aqueous solution of 2 µM calcein-AM was prepared and added to the apical compartment after the cultures were washed twice with PBS. After incubation for 15 min at 37 C, the stain was removed, and the cultures were washed once. Fresh PBS was then added to the apical compartment to prevent drying out as the samples were imaged. Tiled images of the Transwell membranes were then analyzed via mean fluorescent intensity using analysis tools in ImageJ.

### Oxygen measurements

Oxygen tension in both apical and basolateral compartments was monitored using Lucid Scientific’s ruthenium based optical probes and logging software^28^. The two probes were fixed 3 mm from the apical surface and at the same depth as the basolateral surface of the cell culture insert membrane, respectively. Oxygen tension measurements were continuously collected in 5.1 s intervals during the 9 h overnight periods.

### Bacterial growth

The bacterial numbers in the co-cultures were measured via spotting of the apical and basolateral media on fastidious anaerobe agar plates incubated in an anaerobic chamber and counting the colonies that arose. The timepoints chosen for microbe spotting were 2 h, 17 h, 26 h, 41 h, and 50 h after bacterial inoculation, as those corresponded, respectively, to immediately after the inoculation period, the end of the first overnight period, experimental midpoint, end of the second overnight period, and experimental conclusion. At the conclusion of experiments, following the collection of the apical media, the monolayer was scraped from the Transwell® membrane and homogenized in 300 µL of sterile apical media. This homogenate was then plated on agar to determine the concentration of bacteria that attached to the mammalian cells over the course of the experiment. All samples were diluted in sterile PBS to ensure plates could be accurately counted.

### Lipopolysaccharide measurement

The concentration of LPS in the apical compartment was measured using a *Limulus* amebocyte lysate (LAL) chromogenic endotoxin quantification kit (Pierce). The assay was performed according to the manufacturer’s procedure. In brief, 50 µL of centrifuged and filtered sample media and known standards were added to a 96 well plate kept at 37 C. These samples were incubated with 50 µL of LAL reagent for 10 min and 100 µL of chromogenic solution for 6 min at 37 C. After incubation, 100 µL of stop solution (25% acetic acid) was added to each well, and the absorbance was then measured at 405 nm. The concentration of LPS was determined from a standard curve using LPS isolated from *E. coli* O111:B4 as a standard. LPS measurements were conducted using media samples collected at the same timepoints as described above for analysis of bacterial growth.

### Reactive oxygen species measurement

Reactive oxygen species (ROS) measurements were performed via a chemiluminescent Acridan assay modified from the procedure described by Uy et al.^29^ which measures a broad spectrum of reactive species, such as peroxides, superoxide, and oxygen and peroxy radicals, although it cannot specify the exact species being produced. Briefly, 50 µL of the apical media samples and 50 µL of PBS were combined in a 96 well plate. Next, solutions A and B from the G.E. Healthcare Amersham ECL Plus Western Blotting Detection Reagent kit were mixed in a 40:1 ratio as specified by the manufacturer; 50 µL of this mixed detection solution was then added to each sample, and the plate was incubated in the dark for 5 min at room temperature. The plate’s luminescence was read on a Biotek Synergy 2 plate reader, and the concentration of ROS determined using a standard curve obtained by measuring the luminescence of hydrogen peroxide in an excess of horse radish peroxidase (Sigma) incubated with the detection solution as described by Zhu et al.^30^. Measurements were taken within 45 min of media collection to minimize ROS decomposition^29^. The effect of antioxidants on the concentration of ROS in the collected media was explored by adding 3, 5, and 10 mM of N-acetyl cysteine to the collected media sample-PBS mixture 30 min prior to adding the detection solution. ROS analyses were conducted using media samples collected at the same timepoints as described above for analysis of bacterial growth.

### Nitrite assay

The concentration of nitrites was measured in the collected media as a surrogate for reactive nitrogen species (RNS) concentration using a Griess reagent kit from Abcam®. The measurements were carried out according to the manufacturer’s instructions. Briefly, medium samples were combined with the Griess reaction mix (Griess Reagent I and II, nitrite assay buffer) and incubated for 10 min before measuring the absorbance of the samples at 540 nm. Concentrations of nitrite were calculated according to a standard curve generated from the included nitrite solution. Nitrite concentration measurements were conducted using media samples collected at the same timepoints as described above for analysis of bacterial growth.

### Lipid peroxidation assay

Lipid peroxidation is an indicator of ROS-mediated cell damage, and was measured using a kit from Abcam® for the detection of malondialdehyde (MDA), a commonly used biomarker for lipid peroxidation in cell media^31^. In brief, apical media samples and standards were placed in a 96-well plate and incubated for 30 min at room temperature with the MDA color reagent, and then incubated for another 60 min with the reaction solution. The 96-well plate was read at 695 nm absorbance, and the concentration of MDA in the samples was calculated using a standard curve generated from the included MDA solution. Lipid peroxidation analyses were conducted using media samples collected at the same timepoints as described above for analysis of bacterial growth.

### Alcian Blue quantification of secreted mucin

The concentration of secreted mucin in apical medium samples was measured via a colorimetric assay using Alcian Blue solution (Sigma)^19,32^. Briefly, apical media samples were mixed with the Alcian Blue solution in a 3:1 ratio for 2 h in a 96-well plate. After, samples were centrifuged at 1870 × *g* for 30 min at 20 degrees Celsius, the supernatant removed, and the plate washed with a solution of 40% (v/v) ethanol/0.1 M sodium acetate buffer containing 25 mM MgCl_2_ at pH 5.8. After washing, the plate was centrifuged again with the same parameters and the supernatant removed. Pellets were then dissolved in a 10% (v/v) solution of sodium dodecyl sulfate (Sigma) and absorbance measured at 620 nm. Standards were prepared by dissolving type II mucin from porcine stomach (Sigma) in water and their absorbance measured in parallel with the samples.

### Cytokine ELISA

To provide insight into the inflammation state of the basolateral and apical compartments, ELISAs for tumor necrosis factor alpha (TNF-α) (Abcam®) and interleukin 1 beta (IL-1β) (Abcam®) were carried out using medium collected from microbial-epithelial co-cultures at 5, 29, and 47 h that had been centrifuged at 6000 rpm for 5 min to pellet any microbes or cellular debris, then filtered through 0.22 µm mesh filters, and stored at -80 C until use. Both assays were carried out according to manufacturer instructions. In brief, for the TNF-α assay, 50 µL of standards and samples were co-incubated with 50 µL of a 1:1 cocktail of capture and detector antibodies in pre-coated wells. After incubation and washing each well thrice, a development solution was added and the plate was again incubated in the dark for 12 min, after which the stop solution was added. After incubating for 1 min, absorbance was read at 450 nm. The IL-1β assay was performed by incubating 50 µL of standards and samples in pre-coated wells for 2 h, then washing 5 times, adding a biotinylated antibody, and incubating again for 2 h. After washing each well 5 times, a Streptavidin peroxide conjugate was added to each well and incubated for 30 min, followed by a 12-min incubation with a chromogen solution, and finally the addition of the stop solution followed immediately by reading the plate at 450 nm.

### Statistical analysis

Statistical analysis was conducted via ANOVA and post-hoc multiple comparisons via Tukey’s honestly significant difference criterion for analyzing differences between groups at the same timepoint or within a group at more than two timepoints. Paired sample t-tests were used to determine differences within the same group at two timepoints. All statistical calculations were carried out in MATLAB®.

## Results

### Microbial impact on monolayer integrity is species dependent

**Figure 1:**
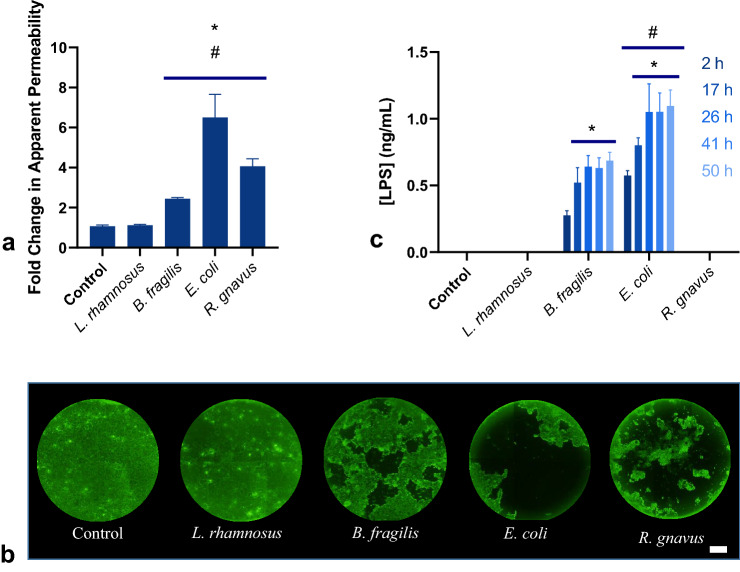
Co-culture of in vitro intestinal mucosa with individual bacterial species results in severe monolayer damage for all co-cultures excluding those co-cultured with *L. rhamnosus*. (**a**) Fold change in apparent permeability of monolayers after 50 h of co-culture [n = 10 samples from 5 independent experiments, * = Significant difference compared to control (*B. fragilis p* = 0.02, *E. coli, R. gnavus p* < 0.0001), # = Significant difference compared to *L. rhamnosus* co-cultures (*B. fragilis p* = 0.04, *E. coli, R. gnavus p* < 0.0001)]. (**b**) Representative Calcein AM stained monolayers after 50 h co-culture (Scale bar = 1 mm). (**c**) Lipopolysaccharide concentration measured in apical media [n = 10 samples from 5 independent experiments, * = Significant difference compared to 2 hour timepoint within the same group (*B. fragilis* *p* = 0.03 to < 0.0001, *p* = 0.03 to < 0.0001, *E. coli p* = 0.01 to < 0.0001), # = Significant difference compared to *B. fragilis* at the same timepoint (p = 0.03 to < 0.0001)].

The epithelial-immune cultures were exposed in the apical compartment to 6.67 × 10^4^ CFU per mL of one of four bacterial species: *L. rhamnosus* KLE2101*, B. fragilis* KLE1958*, E. coli* MG1655*,* or *R. gnavus* KLE1940, which were selected based on their presence in the human commensal gut microbiota and the variety of oxygen requirements, motility, and Gram status that they represent (Table 1). The bacteria were added to the apical medium and incubated for 2 h to allow for attachment, after which the apical and basolateral media were completely replaced. The monolayers were then cultured for an additional 48 h with 13 total media changes. Lucifer Yellow permeability tests performed before and after the co-cultures indicated no statistical change in the apparent permeability of monolayers cultured with *L. rhamnosus* compared both to the pre-exposure permeability and the microbe-free control cultures, which had an average apparent permeability of 3.67 × 10^−7^ cm/s, similar to all other groups prior to bacterial exposure (Fig. 1a). The other co-cultures had an average increase in permeability ranging from 2.4 to 6.5 times their original value. Likewise, transepithelial electrical resistance (TEER) measurements showed steady values pre- and post-experiment for the control and *L. rhamnosus* groups, while the three other groups displayed dramatic decreases in TEER following microbial incubation (Fig. 1, S. Fig. 1B). Staining of the monolayers with calcein AM supported the correlation of increased permeability with monolayer damage, with similar staining patterns for the control and *L. rhamnosus* groups, while the other three groups were heavily damaged, with extensive delamination of the monolayer and loss of fluorescent signal (Fig. 1b, S. Fig. 1A). It is noted that *L. rhamnosus* is considered a probiotic species as it has been found to promote cell growth and wound healing, and is in fact sold as a commercial supplement^16,33^.

**Table 1:** Summary of bacterial characteristics by species^36,50,51,54^.

*L. rhamnosus* KLE2101	*B. fragilis* KLE1958	*E. coli* MG1655	*R. gnavus* KLE1940
Gram positive	Gram negative	Gram negative	Gram positive
Non-motile	Non-motile	Motile	Motile
Facultative anaerobe	Obligate anaerobe	Facultative anaerobe	Obligate anaerobe

Measurement of LPS levels in the apical media over the culture period reflected the expected production of endotoxin by the two gram-negative species, *E. coli* and *B. fragilis* (Fig. 1c). Endotoxin levels increased over time for both groups, with *E. coli* producing higher levels of LPS than *B. fragilis* in culture at each timepoint, measuring at a maximum of 1.1 ng/mL versus 0.7 ng/mL, respectively. These concentrations represent the high end of the range reported for blood plasma under physiological conditions and of the range previously reported as physiologically relevant for analysis of LPS effects on enterocytes (0–1 ng/mL)^34,35^. The control, as well as the two gram-positive species *L. rhamnosus* and *R. gnavus*, did not have any measurable LPS in the apical media, also as expected.

### All bacterial species achieve similar levels of growth during co-culture

**Figure 2:**
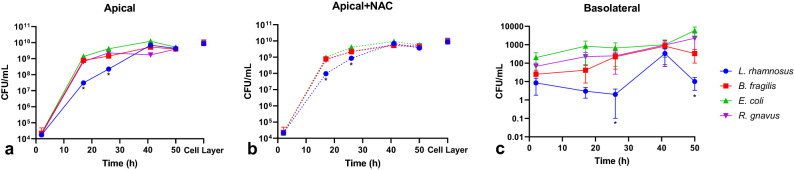
Bacterial growth was similar in magnitude for all groups and unaffected by antioxidant supplementation. Bacterial concentration in apical media (**a**) and NAC supplemented apical media (**b**) collected at various timepoints of the experiment and from the cell layer at the conclusion of the 50 h co-culture. (**c**) Bacterial concentration in basolateral media collected at various timepoints of the experiment. [n = 10 samples from 5 independent experiments, * = Significant difference compared to all other groups (*L. rhamnosus p* = 0.04 to < 0.0001)].

During the co-culture period, the bacterial growth was monitored by periodically plating the media samples on fastidious anaerobe agar plates and incubating the agar plates in an anaerobic chamber. Additionally, cell layers were homogenized to determine the number of bacteria attached to the monolayer at the conclusion of the 50 h experiment. During the course of the experiment, there were no bacteria detected in the apical or basolateral compartments of control cultures. All of the bacterial species were present at similar concentrations at early (2 h) and later (41 and 50 h) time points in culture, and similar numbers of adherent bacteria were measured on the cell layer at the conclusion of the experiments in the different experimental groups (Fig. 2a). The number of bacteria adherent on the cell layer was also similar to the number of bacteria in the apical media at 50 h, suggesting that supernatant measurements served as suitable surrogates for monitoring bacterial load at the epithelial surface (Fig. 2a). However, the concentration of *L. rhamnosus* in the apical media was 20–40 times lower than the concentrations of the other microbes when measured after 17 h of co-culture, and 6–18 times lower when measured after 26 h of co-culture. This difference in growth may be due to inherent differences in growth kinetics and the lag phase of *L. rhamnosus* compared to the other microbes, or perhaps due to a relative inefficiency of consuming mucins and mucus sugars when compared to the other species, as it is noted that *B. fragilis* and *R. gnavus* are voracious consumers of mucus components^36,37^. It is also possible that this somewhat delayed growth contributed to the improved monolayer integrity of the *L. rhamnosus* co-cultures when compared to the other experimental groups. The microbe concentrations had very little change from 41 to 50 h, peaking at 10^10^ CFU per mL.

Relatively low and constant concentrations of bacteria were measured in basolateral media in all groups other than control (Fig. 2c). While detection of bacteria in the basolateral media indicated breaching of the epithelial barrier, the basolateral microbe concentrations ranged from approximately 3 to 9 orders of magnitude lower than apical counterparts, in spite of the relative abundance of nutrients in the basolateral medium, possibly due to a combination of frequent medium changes and the barrier properties of the cellular monolayers. Notably, *L. rhamnosus* concentrations were lower than the other experimental groups, which was likely associated with and contributed to the reduced monolayer damage when compared to the other experimental groups.

### Bacterial co-culture is capable of reducing apical oxygen levels near the monolayer surface over time

**Figure 3:**
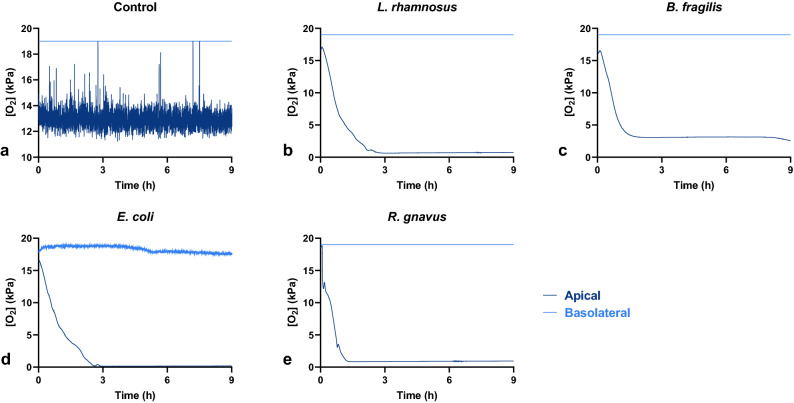
Co-culturing an intestinal mucosa model with live microbes results in a decline in measured oxygen levels compared to control. Representative traces of oxygen tension in the apical and basolateral compartments over the course of a 9-h overnight period for each co-culture. Measurements repeated a total of three times over three independent experiments.

The addition of bacteria led to a reduction in apical oxygen levels measured 3 mm from the monolayer surface. In the absence of bacteria, oxygen levels fluctuated between 14 and 11 kPa over the course of the first 9 h overnight period (Fig. 3). In contrast, the addition of bacteria, including obligate anaerobes *B. fragilis* and *R. gnavus*^36^, reduced oxygen concentration over time from saturated levels to less than 1 kPa. This suggests that bacteria-epithelium co-cultures are capable of establishing low oxygen environments appropriate for the propagation of bacteria intolerant to aerobic culture conditions. This finding is corroborated by a report from Zamora et al., which used an identical epithelial-immune culture system to co-culture the microaerophile *C. jejuni*^19^. The mechanism by which this occurs is unclear, although it is possible that microbial products sensed by the epithelial cells drive consumption of oxygen. For example, it has been noted that butyrate produced by some commensals interacts with epithelial PPAR-γ receptors to maintain low luminal oxygen levels in mice and in Caco-2 cell culture models^38^. As other bacteria-epithelium co-cultures have been conducted at atmospheric oxygen^12–19^, apical oxygen levels may likely have been reduced in these studies, as well.

In contrast, there was little change in basolateral oxygen, which remained near saturated levels over the measurement period. These patterns of near saturated basolateral oxygen and reduced apical oxygen levels were repeated in traces that tracked oxygen tension for the 48-h period following bacterial attachment (S. Fig. 2). The apical oxygen would decrease over the time course between medium changes and then spike back to saturated levels following the media change, meanwhile the basolateral oxygen tension would essentially remain steady near saturation or decrease only slightly between medium changes. The survival of the mammalian monolayers (in particular in the *L. rhamnosus* group, which demonstrated barrier and monolayer integrity throughout culture) in these conditions suggests that the cultures are able to receive sufficient oxygen from the basolateral compartment to prevent hypoxia-related damage. It is noted that the changing oxygen environment and transient high oxygen exposure likely affects bacteria viability and function, motivating future investigation of the impact of initial and transient oxygen concentration on bacterial activity in model gut systems.

### Culture of epithelial-immune monolayers with microbe conditioned media increases permeability

**Figure 4:**
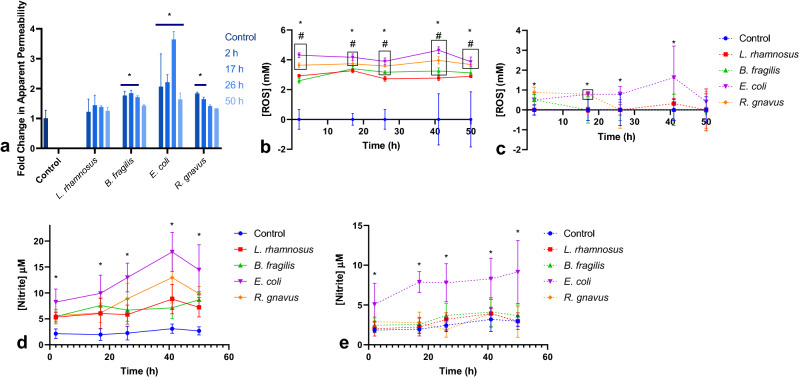
Spent medium from microbe co-cultures increases monolayer permeability due in part to elevated ROS and resultant RNS, which can be reduced via NAC supplementation. (**a**) Fold change in monolayer apparent permeability, measured via Lucifer Yellow, after 24 h exposure to conditioned media collected at 2, 17, 26, and 50 h relative to controls incubated in fresh media [Control n = 6, *L. rhamnosus, B. fragilis, E. coli, R. gnavus* n = 2, * = Significant difference compared to control (*B. fragilis p* values range from ns to *p* = 0.03, *R. gnavus p* values range from ns to *p* = 0.03, *E. coli p* values range from *p* = 0.002 to *p* < 0.0001)]. (**b**) Reactive oxygen species concentration in apical media over the course of 50 h of co-culture [n = 10 samples from 5 independent experiments, * = Significant difference of all co-culture groups compared to control (All groups *p* < 0.0001), # = Significant difference of boxed groups compared to *L. rhamnosus* co-cultures (*B. fragilis p* value ranges from ns to *p* = 0.03, *E. coli p* value ranges from *p* = 0.002 to *p* < 0.0001, *R. gnavus p* value ranges from *p* = 0.02 to *p* < 0.0001)]. (**c**) Reactive oxygen species concentration with NAC supplementation of the apical media (n = 10 samples from 5 independent experiments, * = Significant difference compared to control (*E. coli p* value ranges from ns to *p* < 0.0001, *R. gnavus* ranges from ns to *p* = 0.04, *L. rhamnosus, B. fragilis* ns), boxed group shares significance). (**d**) Nitrite concentration in apical media over the course of 50 h of co-culture (n = 6 samples from 4 independent experiments, * = Significant difference of all bacterial co-culture groups compared to control (*L. rhamnosus p* values range from *p* = 0.04 to *p* = 0.0008, *B. fragilis p* values range from *p* = 0.02 to *p* = 0.0004, *R. gnavus p* values range from *p* = 0.03 to *p* < 0.0001, *E. coli p* values range from *p* = 0.0003 to *p* < 0.0001). (**e**) Nitrite concentration with NAC supplementation of the apical media [n = 6 samples from 4 independent experiments, * = Significant difference compared to control (*E. coli p* values range from *p* = 0.006 to *p* < 0.0001)].

To determine if a factor other than contact with live bacteria was contributing to the observed monolayer damage, epithelial-immune cultures were exposed to media collected from the microbial co-cultures at various timepoints and passed through a 0.22 µm filter to remove the bacteria. Monolayers were incubated with these conditioned media samples on the apical sides of the monolayers for 24 h. Lucifer Yellow permeability measurements performed before and after the conditioned media exposure indicated that the conditioned media from *B. fragilis, E. coli* and *R. gnavus* was sufficient to increase the monolayers’ apparent permeability compared to control and to the *L. rhamnosus* spent media group (Fig. 4a). The significance of the observed response was dependent on the time in culture at which the spent media was collected. The *E. coli* conditioned media led to significant permeability increases with media collected at 17, 26, and 50 h compared to both control cultures and cultures incubated with *L. rhamnosus* conditioned media. Incubation with *R. gnavus* conditioned media led to an increase in permeability with media collected at the 2 h timepoint compared to *L. rhamnosus*, and exposure to media collected at the 2 h and 17 h timepoints produced significant increases in permeability compared to control. *B. fragilis* spent media likewise elicited a permeability increase compared to control with samples collected from the 2, 17, and 26 h timepoints.

### Reactive oxygen species (ROS) and nitrite concentrations were elevated in apical media, but reduced with N-acetyl cysteine

It was hypothesized that the accumulation of reactive oxygen species (ROS) was contributing to the observed monolayer damage, due to their known production by dendritic and epithelial cells in vivo in the intestine in response to bacterial factors, such as ligands for NOD-like receptors^22,27^. It was observed that the ROS concentration was significantly elevated (*p* < 0.0001) in all media collected from the bacterial groups at measured timepoints (2, 17, 26, 41, and 50 h of culture) compared to the control group (Fig. 4b). It is also notable that the co-cultures with *L. rhamnosus* had significantly lower ROS levels than other bacterial groups at multiple time points, which likely played a role in the improved survival of those co-cultures when compared to the other cohorts. Lactobacilli, like *L. rhamnosus,* are actually themselves capable of producing H_2_O_2_, thereby consuming oxygen, which could deplete the oxygen available for producing other, more reactive species^29,39^. It is noted that ROS levels measured in this study are likely elevated due to the high oxygen in vitro culture environment and the known effect of oxygen on ROS production *in vitro*^40–42^*.* Thus, future experiments may explore the impact of lower physiological oxygen levels on measured ROS generation.

Altering the apical media formulation via the addition of 3 mM of the antioxidant N-acetyl cysteine (NAC), a common antioxidant used in primary intestinal cultures^43–46^, resulted in drastically reduced ROS concentration for all co-cultures over the course of the experiment. Maximum average ROS levels in the absence of NAC ranged from 4.4 to 3.4 mM across each of the four experimental groups. In the presence of 3 mM NAC, these values were reduced to a range of 1.6 mM to 330 µM (Fig. 4c). Supplementation with NAC did not have a notable impact on the bacterial growth, both in total and at each time point (Fig. 2b).

Medium nitrite concentration was also measured as an indicator of reactive nitrogen species (RNS) concentration. Like ROS, RNS can be generated by dendritic cells and play important roles in response to bacterial factors, and indeed are produced from the reaction of superoxide and NO^47,48^. Similar to the measured ROS, nitrite levels were elevated compared to controls for all bacterial co-cultures when the apical medium was not supplemented with NAC, although the concentrations of nitrites were lower than those of ROS (Fig. 4d). Likewise, NAC supplementation of the apical medium was able to reduce the concentration of nitrites measured in the medium to control levels for all co-culture groups other than *E. coli* co-cultures, which were reduced, but still significantly higher than control (Fig. 4e). RNS was likely reduced by NAC supplementation through the reduction of ROS, which themselves contribute to RNS production^47,48^.

### Supplementation of NAC in the apical media has a positive effect on monolayer viability for B. fragilis and R. gnavus co-cultures

**Figure 5:**
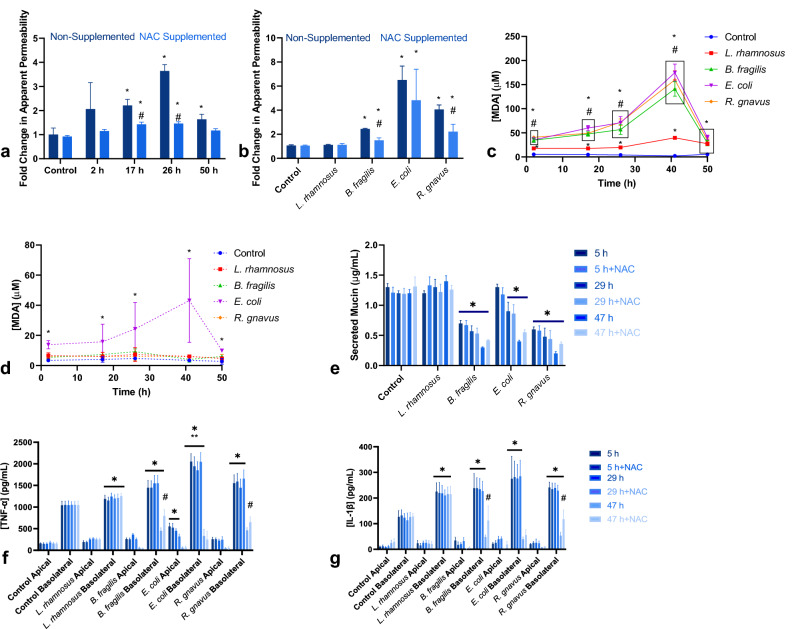
Apical supplementation of NAC reduces MDA and the degree of change in monolayer permeability compared to non-supplemented co-cultures. (**a**) Fold change in apparent permeability of monolayers cultured for 24 h with *E. coli* spent medium collected from various time points of co-culture experiments, with NAC supplementation and without NAC supplementation [n = 2, * = Significant difference compared to control (17 h *p* values range from *p* = 0.04 to *p* = 0.02, 26 h *p* values range from *p* = 0.04 to *p* < 0.0001, 50 h *p* = 0.04), # = Significant difference compared to Non-Supplemented samples collected from same timepoint (17 h *p* = 0.02, 26 h *p* = 0.009)]. (**b**) Effect of NAC supplementation on the change in apparent permeability of single-species co-cultures after 50 h [n = 10 over 5 independent experiments, * = Significant difference compared to control (*B. fragilis p* = 0.02, *E. coli, R. gnavus p* < 0.0001), # = Significant difference compared to Non-supplemented samples (*B. fragilis p* = 0.03, *R. gnavus p* = 0.001)]. (**c**) Concentration of MDA in apical media over the course of 50 h of co-culture [n = 10 over 5 independent experiments, * = Significant difference compared to control (*L. rhamnosus p* values range from *p* = 0.003 to *p* < 0.0001, *B. fragilis, E. coli, R. gnavus p* < 0.0001), # = Significant difference compared to *L. rhamnosus* co-cultures (*B. fragilis, E. coli, R. gnavus p* values range from ns to *p* < 0.0001), boxed groups share significance]. (**d**) Concentration of MDA with NAC supplementation of apical media [n = 10 over 5 independent experiments, * = Significant difference compared to control (*E. coli p* values range from *p* = 0.01 to *p* < 0.0001)]. (**e**) Concentration of apically secreted mucin measured from medium collected at 5, 29, and 47 h after bacterial inoculation [n = 6 over 3 independent experiments, * = significant difference compared to control values from corresponding timepoints *(B. fragilis, R. gnavus p* < 0.0001, *E. coli p* values range from *p* = 0.03 to *p* < 0.0001)]*.* (**f**) Concentration of TNF-α in the apical and basolateral chambers at multiple time points with and without NAC supplementation. [n = 4 across 2 independent experiments, * = significant difference compared to the corresponding control value, ** = significant difference compared to corresponding *L. rhamnosus* value, # = significant difference compared to the corresponding non-supplemented value. (*L. rhamnosus p* values range from *p* = 0.04 to *p* = 0.02, *B. fragilis*, *E. coli*, *R. gnavus p* < 0.0001)]. (**g**) Concentration of IL-1β in the apical and basolateral chambers at multiple time points with and without NAC supplementation. [n = 4 across 2 independent experiments, * = significant difference compared to the corresponding control value, # = significant difference compared to the corresponding non-supplemented value. (*L. rhamnosus p* values range from *p* = 0.03 to *p* = 0.01, *B. fragilis*, *E. coli*, *R. gnavus p* < 0.0001)].

To test the feasibility of using NAC to reduce the impact of oxidative damage, spent media collected from *E. coli* co-cultures was supplemented with NAC. The addition of NAC reduced the impact of the spent media on cell monolayers (Fig. 5a). While permeability was still significantly increased with exposure to media collected after the first overnight and midpoint periods when compared to controls, the increase in permeability was significantly reduced, for example from over 260% to roughly 45% in media collected at the experimental midpoint (Fig. 5a).

Despite this promising pilot result however, supplementation of media with NAC in co-cultures with live bacteria did not uniformly reduce the impact of bacterial co-culture on permeability (Fig. 5b), in spite of reduction in ROS (Fig. 4b). Permeability of control and *L. rhamnosus* cultures were not affected, and there was no statistically significant effect on *E. coli* cultures due to high variability, although, on average, supplemented cultures had lower permeability than non-supplemented cultures. Both *B. fragilis* and *R. gnavus* had significantly lower permeability in cultures with apical NAC compared to those without, although it was still significantly higher than both pre-exposure levels and control cultures (Fig. 5b).

In addition to permeability, the MDA levels were also quantified as a measure of oxidative damage. Bacteria-epithelial co-cultures without NAC had significantly elevated MDA, with levels exceeding 150 µM of MDA released by the cells into the supernatant in instances of monolayer damage and delamination, such as in the *E. coli* and *R. gnavus* groups when measured after the second overnight period (Fig. 5c). The addition of NAC in bacteria-epithelial co-cultures significantly reduced oxidative damage, as reflected by MDA. Antioxidant supplementation was able to bring measured MDA concentrations down to control values in co-cultures with all bacterial species other than *E. coli* (Fig. 5d). NAC treated *E. coli* co-cultures had greater variability in MDA levels than the other experimental groups, reflecting the variability in measured permeability in these cultures (Fig. 5b).

Notably, MDA levels were most elevated following the second overnight period (41 h samples) of the cultures, corresponding to the first visual indications of monolayer damage, such as the appearance of lifted monolayer edges (data not shown), and peak bacterial numbers. This suggests that the extended time without receiving fresh media and removing accumulated bacteria and ROS is a major component contributing to the onset of monolayer damage. Flow of medium during culture likely enables longer-term culture with bacteria^12^ in part through removal of ROS/RNS as well as bacteria and bacterial factors.

**Figure 6:**
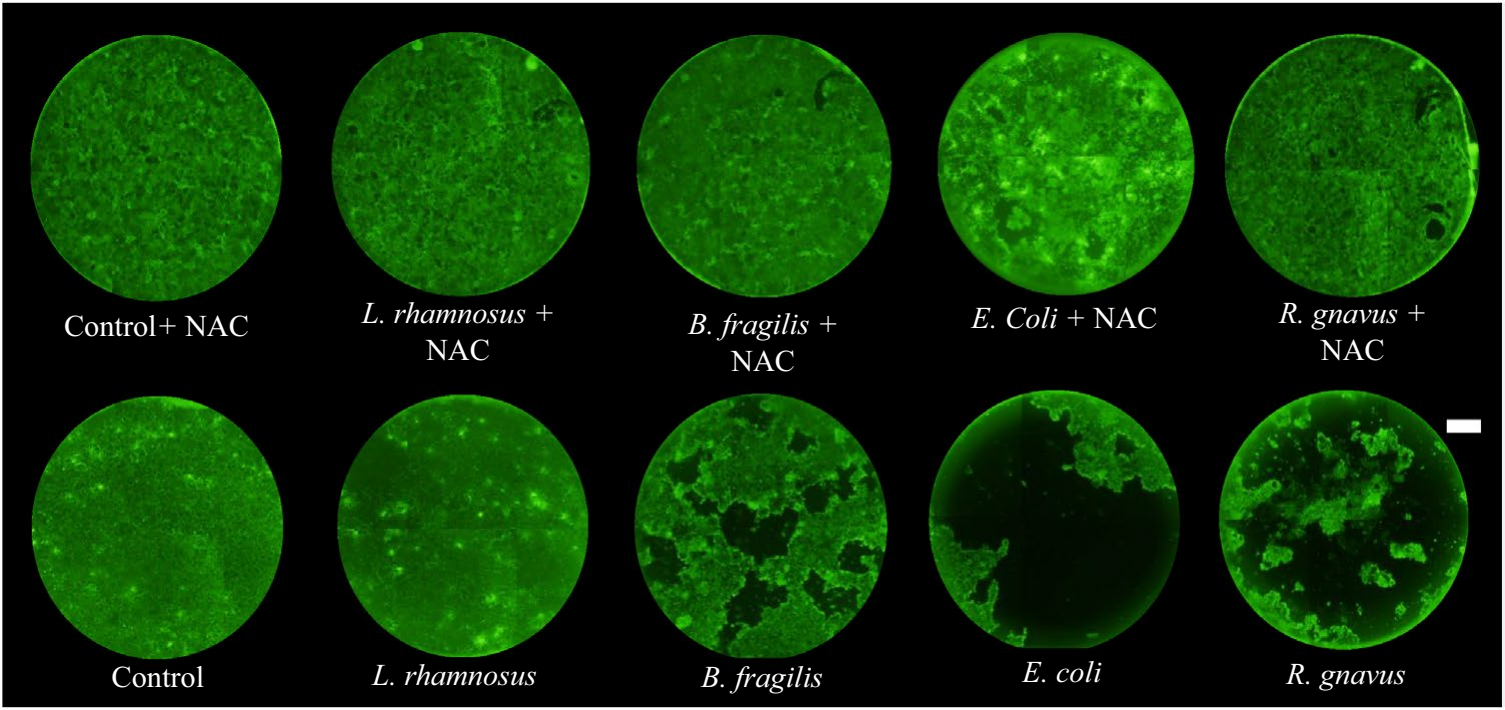
Apical supplementation of NAC improves monolayer viability. Representative Calcein AM stained images of NAC supplemented co-cultures and non-supplemented cultures (scale bar = 1 mm).

Calcein AM staining of the monolayers in cultures supplemented with NAC at the end of the 50 h culture period indicated that the supplementation greatly improved monolayer integrity in *B. fragilis* and *R. gnavus* cultures (Fig. 6). There were few visible holes in the monolayers and no instances of broad delamination as seen in the co-cultures without apical NAC. These results translated to an approximate doubling of quantified mean fluorescent intensities for epithelial layers co-cultured with those two species and supplemented with NAC relative to non-supplemented cultures, although this difference was statistically significant only for *B. fragilis* co-cultures (S. Fig. 1A). TEER also increased dramatically in NAC supplemented *B. fragilis* and *R. gnavus* co-cultures compared to non-supplemented cultures (S. Fig. 1B). Similar to the permeability results, staining patterns in *E. coli* co-cultures supplemented with NAC varied considerably, with certain monolayers comparable to those of treated *B. fragilis* co-cultures, and other monolayers similar to non-treated cultures (Fig. 6).

It is noted that NAC does possess mucolytic properties and can disrupt disulfide bonds between mucins, but when used in this manner it is typically at concentrations more than three times greater than used in this study^49^. Supplementation with NAC did not have a significant effect on measured apically secreted mucin in this study (Fig. 5e). Secreted mucin concentrations in *B. fragilis* and *R. gnavus* co-cultures were significantly reduced relative to control at all timepoints measured, likely due to a combination of the species’ ability to consume mucus components^36,50,51^ and the loss of epithelial cells due to cell death. While not statistically significant, mean mucin for those two groups was higher in NAC supplemented samples at 29 and 47 h, likely due to improved monolayer survival. Mucin production in *E. coli* co-cultures was significantly lower than control at 29 and 47 h, likely due to the loss of epithelial cells. Mucin levels in the control and *L. rhamnosus* co-cultures, as well as in *E. coli* co-cultures at measured timepoints prior to 29 h, were similar to previously published results using the same culture system^19^ (Fig. 5e). The differential consumption of mucin and the significance of mucins in protecting underlying epithelium motivate future studies investigating how consumption of mucin may impact relative position of bacteria with respect to mucus and epithelium. Indeed, as bacteria are present at different radial positions relative to intestinal lumen, mucus, and epithelium in vivo due to differences in oxygen tension and nutrient supply^52^, microbial positioning may significantly impact immune and epithelial responses in vitro.

NAC has also been reported to have an anti-inflammatory effect reflected in cytokine levels^53^, but no impact of NAC supplementation on TNF-α or IL-1β concentrations in apical or basolateral compartments was measured in this study (Fig. 5f, g). TNF-α and IL-1β were chosen for analysis to provide insight into the inflammatory state due to their noted production by dendritic cells in early response to inflammatory stimuli^9,19,20^. Predictably, both cytokines were significantly increased in all microbial groups, with *E. coli* co-cultures displaying the highest average levels, significantly higher than both control and *L. rhamnosus,* at 5 and 29 h. These results are consistent with previous reports with immune-epithelial cultures exposed to bacterial stimulation^19,20^. However, the values for *E. coli, B. fragilis,* and *R. gnavus* co-cultures dropped significantly below control and *L. rhamnosus* values when examined at 45 h, likely due to extensive cell death. It is notable, however, that cytokine levels in NAC supplemented co-cultures with *B. fragilis* and *R. gnavus* at 45 h were significantly increased compared to the non-supplemented cultures, presumably again due to greater numbers of live cells.

Collectively, the finding that the impact of bacterial co-culture on permeability and monolayer integrity was decreased markedly but not completely eliminated with NAC supplementation, paired with the reduction of MDA in most groups to control levels with NAC supplementation, indicates that oxidative damage is a major, but not the solely significant, contributor to mammalian cell damage in co-cultures with bacteria.

## Conclusions

Static co-cultures of single microbial species and mammalian epithelial and immune cells in cell culture inserts were established and maintained over 50 h. The measured growth of bacterial cultures indicate obligate and facultative anaerobes can be maintained in aerobic culture environments in co-culture with host epithelial and immune cells. This is likely due to a microaerobic microenvironment that is created in these cultures. However, the presence of the bacteria has a detrimental effect on the mammalian cells in culture, increasing permeability and causing detachment of epithelial monolayers for microbial species investigated, with the exception of *L. rhamnosus*. Injury to epithelial monolayers is due in part to oxidative damage, which results from the production of reactive species by both epithelial and dendritic cells as part of the host microbe defense. While reduction in oxidative damage was possible with supplementation of NAC, it was not sufficient to eliminate damage (i.e. as reflected by the increased permeability relative to bacteria-free control cultures, and continued presence of small holes in the monolayers) in *B. fragilis, E. coli,* or *R. gnavus* co-cultures. However, supplementation with NAC did significantly improve monolayer health, as indicated by the reduced impact of bacterial co-culture on apparent permeability in *B. fragilis* and *R. gnavus* co-cultures, as well as improvements in visible monolayer integrity.

These results suggest a major limiting factor in long-term static microbe-mammalian co-culture is oxidative damage, and emphasize that additional factors contribute to in vitro homeostasis of microbial-mammalian co-cultures, such as signaling from microbial factors and inflammatory immune responses. Flow conditions likely reduce local concentrations of molecules that cause oxidative stress, as well as other microbial and inflammatory factors, to enable longer term bacteria-epithelium homeostasis in culture.

## Supplementary Information

Supplementary Figure 1.

Supplementary Figure 2.
