# Supplementary material for: Reactive oxygen species limit intestinal mucosa-bacteria homeostasis in vitro
Source: Sci Rep. 2021 Dec 9;11:23727. doi: 10.1038/s41598-021-02080-x (PMC8660821; doi:10.1038/s41598-021-02080-x)
Supplement: Supplementary file 1 — Supplementary Figure 1. [file 41598_2021_2080_MOESM1_ESM.pdf]

Supplemental Figure 1

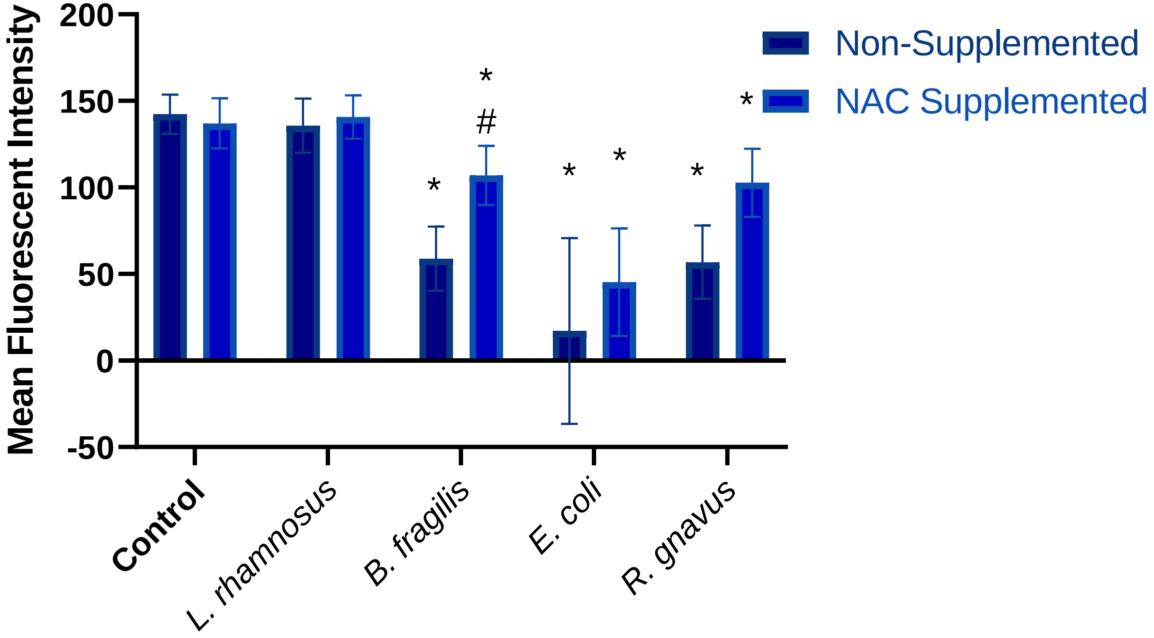

a

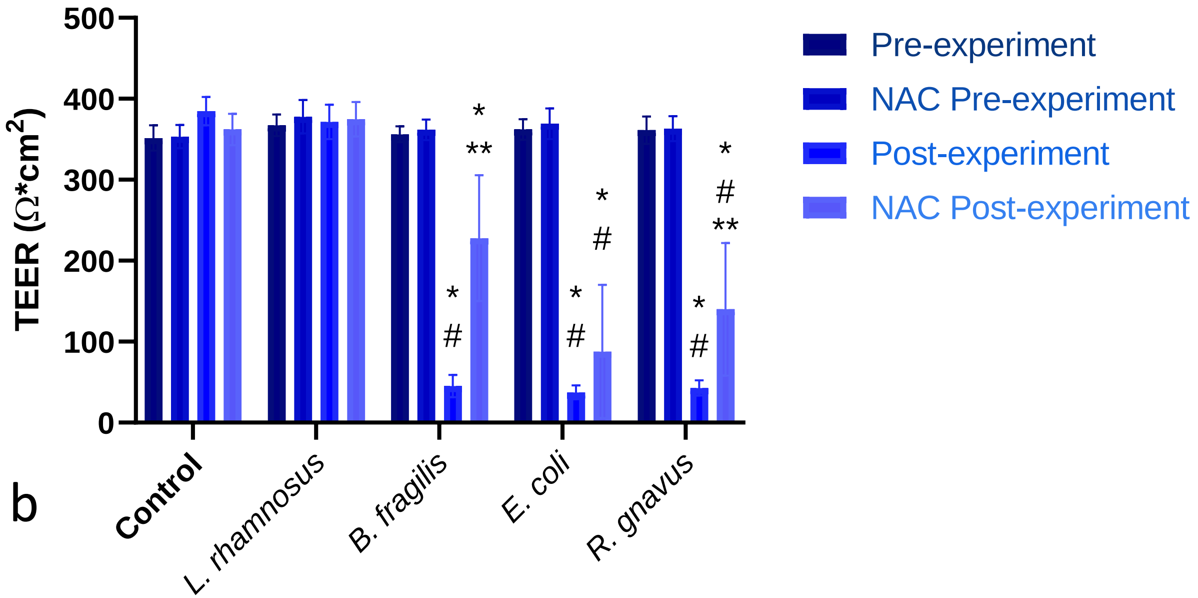

b

Supplementary figure 1: a. Mean fluorescent intensity of calcein AM monolayer images [n=4 over 3 independent experiments * = Significant difference compared to control, # = Significant difference compared to non-supplemented sample ($p < 0.05$)].

b. Transepithelial electrical resistance (TEER) measured prior to, and following the 50-hour microbial co-culture period in non-supplemented and NAC-supplemented cultures. TEER significantly decreases in the *B. fragilis, E. coli* and *R. gnavus* groups both compared to control and to pre-experimental values. [n=10 over 5 independent experiments * = Significant difference compared to control, # = Significant difference compared to pre-experiment sample, ** = Significant difference compared to corresponding non-supplemented samples ($p < 0.05$)].
