# Supplementary material for: Reactive oxygen species limit intestinal mucosa-bacteria homeostasis in vitro
Source: Sci Rep. 2021 Dec 9;11:23727. doi: 10.1038/s41598-021-02080-x (PMC8660821; doi:10.1038/s41598-021-02080-x)
Supplement: Supplementary file 2 — Supplementary Figure 2. [file 41598_2021_2080_MOESM2_ESM.pdf]

Supplemental Figure 2

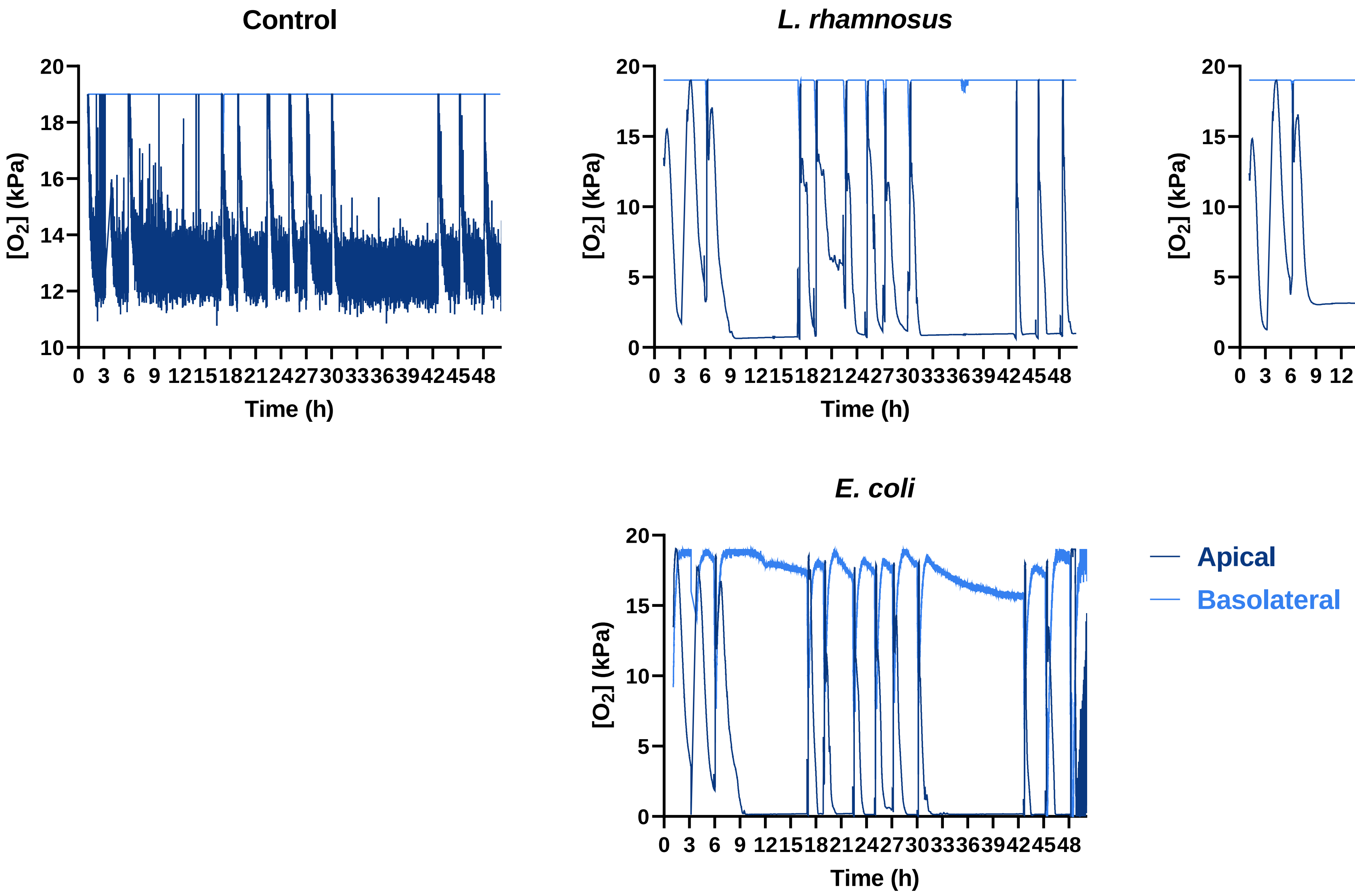

Supplementary figure 2: Traces of oxygen tension over the course of 48 hours following bacterial attachment for *L. rhamnosus, B. fragilis,* and *E. coli* co-cultures as well as a microbe free control.
